# Supplementary material for: Effects of L-carnitine supplementation for women with polycystic ovary syndrome: a systematic review and meta-analysis
Source: PeerJ. 2022 Sep 16;10:e13992. doi: 10.7717/peerj.13992 (PMC9484467; doi:10.7717/peerj.13992)
Supplement: Supplemental Information 2 [file peerj-10-13992-s002.docx]

**Appendix 1**

**Cochrane Central**

1 Polycystic Ovarian Syndrome Or PCOS

2 L Carnitine

3 #1 and #2

4 Polycystic Ovarian Syndrome OR PCOS in Title Abstract Keyword AND L Carnitine in Title Abstract Keyword (Word variations have been searched)

5 (Polycystic Ovarian Syndrome OR PCOS):ti,ab,kw AND (L Carnitine):ti,ab,kw (Word variations have been searched)

**Pub Med**

1 Polycystic Ovarian Syndrome Or PCOS

2 L Carnitine

3 1 and 2

4 PCOS:ti,ab,kw AND L Carnitine:ti,ab,kw (Word variations have been searched)

**Other databases (CINAHL, PsyInfo)**

1 Polycystic Ovarian Syndrome Or PCOS

2 L Carnitine

3 1 and 2

4 PCOS:ti,ab,kw AND L Carnitine:ti,ab,kw (Word variations have been searched)
