# Supplementary material for: Effects of L-carnitine supplementation for women with polycystic ovary syndrome: a systematic review and meta-analysis
Source: PeerJ. 2022 Sep 16;10:e13992. doi: 10.7717/peerj.13992 (PMC9484467; doi:10.7717/peerj.13992)
Supplement: Supplemental Information 3 [file peerj-10-13992-s003.pdf]

## 1. The rationale for conducting the systematic review/meta-analysis

L-carnitine plays a significant role in the metabolism of glucose and oxidative stress. There has never been a thorough evaluation or critical appraisal of the literature in this field which include the metabolic profile and the fertility outcomes. Many methodological aspects of a study might raise the likelihood of bias, and hence the chance that the results are erroneous, in addition to task demands and response bias. Therefore, this meta-analysis and systematic review is appraised the evidence and provided the summary evidence regarding the use of l-carnitine to improve the clinical pregnancy outcome and improving the metabolic markers in patients with PCOS.

Information on L carnitine supplementation's effect might help the physicians choose and decide on the alternative supplementation to increase the clinical pregnancy rate and improve metabolic markers in PCOS.

## 2. The contribution that it makes to knowledge considering previously published related reports, including other meta-analysis and systematic reviews

To evaluate the effect of LC on PCOS patients, we conducted a comprehensive literature study. From nine trials, only five trials can be sub grouped into similar combination of comparison in which two trials (Ismail *et al.*, 2014; Kortam *et al.*, 2020) in Comparison 1 for the outcome of clinical pregnancy rate and ovulation rate, and three trials (Jamilian *et al.*, 2017; Samimi *et al.*, 2016; Talari *et al.*, 2019) in Comparison 4 for BMI outcome. Thus, as a result, the application of the findings in this review is limited. On the outcome basis, three primary outcomes: clinical pregnancy rate, ovulation rate, and FPG have similar trials with similar combination of comparisons, in which two trials in clinical pregnancy rates, two trials in ovulation rate, and three trials in FPG. The overall quality of the evidence contributing to this review is moderate to low. The type of comparison and supplementation dosage varied among the trials. We can only do meta-analysis for particular comparisons in most of our meta-analyses since there are not enough trials with similar combinations of comparisons. We ran into high heterogeneity in the meta-analysis, and we could not segment any further since there were not enough trials in each group comparison. We aimed to reduce publication bias by searching different databases without language restrictions and examining the reference lists of all linked articles for additional references. Unfortunately, we cannot guarantee that we have discovered all of the trials in this area. As we have only nine trials included, we could not create a funnel plot to detect bias or heterogeneity, and not all included trials reported similar outcomes. Although the included studies all showed the same direction of effect, we encountered high heterogeneity in our primary outcomes. We could not do subgroup analysis due to limited number of trials.

There was one systematic review has examined the effect of LC in patients with polycystic ovary syndrome (Maleki *et al.*, 2019). They evaluated the potential roles of LC in PCOS patients. It included two observational studies (Celik *et al.*, 2017; Fenkci *et al.*, 2008) and four randomized controlled studies, in which three studies (Ismail *et al.*, 2014; Jamilian *et al.*, 2019b; Samimi *et al.*, 2016) included in this meta-analysis and one study (Latifian *et al.*, 2015) not related to our primary and secondary outcomes. Similar to ours, the BMI had a significant effect on l-carnitine supplementation based on three trials (Ismail *et al.*, 2014; Jamilian *et al.*, 2019b; Samimi *et al.*, 2016), but for lipid profile, one study had a significant effect (Ismail *et al.*, 2014) whereas two studies had insignificance effect (Fenkci *et al.*, 2008; Samimi *et al.*, 2016).

**Highlights of this review**

- L-carnitine supplementation has role in women with polycystic ovarian syndrome.
- BMI and serum LDL, triglyceride, and total cholesterol levels were improved with L-carnitine supplementation.
- Unclear evidence for l-carnitine to improve ovulation and pregnancy rate.
- Side effects of L-carnitine are limited, and more safety data is needed.
