# Supplementary material for: Effects of L-carnitine supplementation for women with polycystic ovary syndrome: a systematic review and meta-analysis
Source: PeerJ. 2022 Sep 16;10:e13992. doi: 10.7717/peerj.13992 (PMC9484467; doi:10.7717/peerj.13992)
Supplement: Supplemental Information 4 [file peerj-10-13992-s004.pdf]

Citation

Mohd Falihin Mohd Shukri, Mohd Noor Norhayati, Salziyan Badrin, Azidah Abdul Kadir. Effects of L-carnitine supplementation for patients with polycystic ovarian syndrome. PROSPERO 2021 CRD42021232433 Available from: https://www.crd.york.ac.uk/prospero/display_record.php?ID=CRD42021232433

Review question

1. How does the L carnitine supplementation give effects on clinical pregnancy rate, ovulation rate, metabolic profile including BMI, lipid profile and fasting blood sugar changes in patient with polycystic ovarian syndrome?

2. How does the L carnitine supplementation give effects on hormonal profile changes and mental health status in patient with polycystic ovarian syndrome?
3. Is L carnitine effective to be used as an adjuvant therapy in patients with polycystic ovarian syndrome?

Searches

We will search trials in the Cochrane Central Register of Controlled Trials (CENTRAL), MEDLINE, EMBASE, Cumulative Index to Nursing and Allied Health Literature (CINAHL) and Psychological Information Database (PsycINFO).We restrict only to English language publications. We will check the reference list of identified RCTs and review articles in order to find unpublished trials or trials not identified by electronic searches. We will search for ongoing trials through the World Health Organization (WHO) International Clinical Trials Registry Platform (ICTRP) https://www.who.int/ictrp/en/ and www.ClinicalTrials.gov.

Types of study to be included

Randomized control trials (RCTs) only comparing L carnitine alone or in combination with clomiphene citrate and/or metformin and/or other dietary supplements with the placebo.

Condition or domain being studied

Polycystic ovary syndrome (PCOS) is a condition which occurs in reproductive-aged women, with the prevalence of approximately 2–20% worldwide. It is characterized by hyperandrogenic anovulation and oligo-amenorrhea, leading to hirsutism, acne, alopecia, increased androgens, irregular menstruation and infertility. Anovulation in PCOS is associated with low follicle-stimulating hormone levels and the arrest of antral follicle development in the final stages of maturation. Medications such as clomiphene citrate, tamoxifen, aromatase inhibitors, metformin, glucocorticoids, gonadotropins, or laparoscopic ovarian drilling can be used for this anovulation problem.

Participants/population

We will include adults age above 18 years old and patients who diagnosed with polycystic ovarian syndrome diagnosed based on the revised European Society for Human Reproduction (ESHRE) and the American Society of Reproductive Medicine (ASRM) diagnosis of PCOS (Rotterdam Criteria 2003).

Intervention(s), exposure(s)

L-Carnitine supplementation alone or in combination with clomiphene citrate and/or metformin, or L carnitine in combination with other dietary supplements regardless duration of therapy.

Comparator(s)/control

Placebo

Main outcome(s)

1. Number of clinical pregnancies by transvaginal ultrasound with a visible intrauterine gestational sac and visible fetal heart rate within duration of intervention

2. Number of ovulations by transvaginal ultrasound with visible leading follicle of ? 18mm within duration of intervention

3. Metabolic profiles changes including:

a) Body mass index (BMI) changes (kg/m2)

b) Serum lipid changes:

LDL (mmol/l, mg/dl)

Triglyceride (mmol/l, mg/dl),

Total cholesterol (mmol/l, mg/dl),

HDL level (mmol/l, mg/dl)

c) Fasting blood sugar (mg/dl)

Measures of effect

We will measure the treatment effect for dichotomous/continuous outcomes using risk ratios (RRs) and absolute risk reduction, and for continuous outcomes we will use mean differences (MDs); both with 95% confidence intervals (CIs).

Additional outcome(s)

1. Hormonal level changes including FSH and LH levels

2. Mental health statuses such as depression score, GHQ score and DASS score (stress, anxiety and depression)

Measures of effect

We will measure the treatment effect for dichotomous/continuous outcomes using risk ratios (RRs) and absolute risk reduction, and for continuous outcomes we will use mean differences (MDs); both with 95% confidence intervals (CIs).

Data extraction (selection and coding) [1 change]

We will use the search strategy to search in the Cochrane Central Register of Controlled Trials (CENTRAL) and MEDLINE (1966 to present) as below:

CENTRAL

1. Polycystic Ovarian Syndrome OR PCOS in Title Abstract Keyword AND L Carnitine in Title Abstract Keyword

2. Polycystic Ovarian Syndrome OR PCOS in Title Abstract Keyword AND Carnitine in Title Abstract Keyword

Pub Med

1. Polycystic Ovarian Syndrome OR PCOS[Title/Abstract]) AND L carnitine[Title/Abstract]

2. Polycystic Ovarian Syndrome OR PCOS[Title/Abstract] AND Carnitine[Title/Abstract]

Searching other resources

We will check the reference list of identified RCTs and review articles to find unpublished trials or trials not identified by electronic searches. We will search for ongoing trials through the World Health Organization (WHO) International Clinical Trials Registry Platform (ICTRP) http://www.who.int/ictrp/en/ and www.ClinicalTrials.gov.

Trial selection

We will search the titles and abstracts from the searches and obtain full-text articles when they have meet the eligibility criteria, or when there is insufficient information to assess the eligibility. We will assess the eligibility of the trials independently and the reasons for exclusion will be documented. Any disagreements between the review authors will be resolved by discussions. We will contact the trial's authors if clarification is needed.

Data extraction

Using data extraction form, from each of the selected trials we will extract:

• study setting

• participant characteristics (age, ethnicity)

• methodology (number of participants randomized and analyzed, duration of follow-up)

• method for diagnosing PCOS

• dose and frequency of the medication;

• clinical pregnancy outcome rate

• number of ovulation rate

• body mass index changes

• serum lipid profile of patient during follow up period particularly in: LDL, Triglyceride, LDL and HDL level

• fasting blood glucose

• hormonal level changes: FSH, LH,

• mental health status example, depression, anxiety and stress

Risk of bias (quality) assessment

We will assess the risk of bias based on random sequence generation, allocation concealment, blinding of participants and personnel, blinding of outcome assessors, completeness of outcome data, the selectivity of outcome reporting and other bias (Higgins 2019). We will resolve any disagreements by discussion.

We assessed the quality of evidence for primary and secondary outcomes according to GRADE methodology (Guyatt 2008) for risk of bias, inconsistency, indirectness, imprecision, and publication bias; classified as very low, low, moderate, or high.

Strategy for data synthesis

We plan to undertake meta-analyses using Review Manager 5.4 software (RevMan 2020) and will use random-effects model to pool data. We plan to use the guide to interpretation of heterogeneity as outlined: 0% to 40% might not be important; 30% to 60% may represent moderate heterogeneity; 50% to 90% may represent substantial heterogeneity; and 75% to 100% would be considerable heterogeneity.

We will assess the presence of heterogeneity in two steps: at face value by comparing populations, settings, interventions, outcomes and secondly, we will assess statistical heterogeneity by means of the I^2 statistic.

We will measure the treatment effect for dichotomous outcomes using risk ratios (RRs) and absolute risk reduction, and for continuous outcomes we will use mean differences (MDs); both with 95% confidence intervals (CIs). Subgroup analysis and investigation of heterogeneity. If possible, we will conduct subgroup analyses on the dosage of L Carnitine

Unit of analysis issues

We will check included trials for unit of analysis errors. Unit of analysis errors can occur when trials randomize participants to intervention or control groups in clusters, but analyze the results using the total number of individual participants. We will adjust results from trials showing a unit of analysis errors based on the mean cluster size and intracluster correlation coefficient (Higgins 2019).

Dealing with missing data

We will contact the original trial authors to request missing or inadequately reported data. We will perform analyses on the available data in the event that missing data are not available.

Sensitivity analysis

We will perform a sensitivity analysis to investigate the impact of risk of bias for sequence generation and allocation concealment of included studies.

Reporting biases

If there are sufficient studies, we will use funnel plots to assess the possibility of reporting biases or small study biases, or both.

Analysis of subgroups or subsets

We will explore the potential sources of heterogeneity. When important heterogeneity is present, we will pool the effect estimate from a fixed-effect and either not pool the studies or use a random-effects model.

Contact details for further information

Salziyan Badrin
salziyan@usm.my

Organisational affiliation of the review

Universiti Sains Malaysia

Review team members and their organisational affiliations

Dr Mohd Falihin Mohd Shukri. School of Medical Sciences, Health Campus Universiti Sains Malaysia
Assistant/Associate Professor Mohd Noor Norhayati. School of Medical Sciences, Health Campus Universiti Sains Malaysia
Dr Salziyan Badrin. School of Medical Sciences, Health Campus Universiti Sains Malaysia
Professor Azidah Abdul Kadir. School of Medical Sciences, Health Campus Universiti Sains Malaysia

Type and method of review

Meta-analysis, Systematic review

Anticipated or actual start date

30 January 2021

Anticipated completion date
31 December 2021

Funding sources/sponsors
None

Conflicts of interest

Language
English

Country
Malaysia

Stage of review
Review Ongoing

Subject index terms status
Subject indexing assigned by CRD

Subject index terms
Carnitine; Dietary Supplements; Humans; Polycystic Ovary Syndrome

Date of registration in PROSPERO
15 March 2021

Date of first submission
11 February 2021

Stage of review at time of this submission

Stage	Started	Completed
Preliminary searches	Yes	No
Piloting of the study selection process	No	No
Formal screening of search results against eligibility criteria	No	No
Data extraction	No	No
Risk of bias (quality) assessment	No	No
Data analysis	No	No

The record owner confirms that the information they have supplied for this submission is accurate and complete and they understand that deliberate provision of inaccurate information or omission of data may be construed as scientific misconduct.

The record owner confirms that they will update the status of the review when it is completed and will add publication details in due course.

Versions
15 March 2021
